# Dynamic changes of autophagic flux induced by Abeta in the brain of postmortem Alzheimer’s disease patients, animal models and cell models

**DOI:** 10.18632/aging.103305

**Published:** 2020-06-13

**Authors:** Zhimin Long, Jingfei Chen, Yueyang Zhao, Wen Zhou, Qiuhui Yao, Yingxiong Wang, Guiqiong He

**Affiliations:** 1 Institute of Neuroscience, Chongqing Medical University, Chongqing 400016, China; 2 Department of Anatomy, Chongqing Medical University, Chongqing 400016, China; 3 Department of Neurorehabilitation, The Affiliated Rehabilitation Hospital of Chongqing Medical University, Chongqing 400016, China; 4 Joint International Research Laboratory of Reproduction and Development, Chongqing Medical University, Chongqing 400016, China

**Keywords:** Alzheimer’s disease, autophagic flux, β-amyloid peptide, transgenic mice, lysosome

## Abstract

Autophagy has been reported to play a dual "double-edged sword" role in the occurrence and development of Alzheimer’s disease (AD). To assess the relationship between AD and autophagy, the dynamic changes of autophagic flux in the brain of postmortem AD patients, animal models and cell models were studied. The results showed that autophagosomes (APs) accumulation and expression of lysosomal markers were decreased in the brains of AD patients. In the brain of APP/PS1 double transgenic mice, APs did not accumulate before the formation of SPs but accumulated along with the deposition of SPs, as well as the level of lysosomal markers cathepsin B and Lamp1 protein decreased significantly. In the brains of APP/PS1/LC3 triple - transgenic mice, the number of APs increased with age, but the number of ALs did not increase accordingly. The activation of autophagy is mainly due to the increase in Aβ rather than the overexpression of mutated APP gene. However, both the treatment with exogenous Aβ_25-35_ and the mutation of the endogenous APP gene blocked the fusion of APs with lysosomes and decreased lysosomal functioning in AD model cells, which may be the main mechanism of autophagy dysregulation in AD.

## INTRODUCTION

Alzheimer’s disease (AD), the main cause of dementia in the elderly, is a complex neurodegenerative disorder [1, 2]. Histopathologically, AD manifests via synaptic abnormalities and neuronal degeneration, as well as extracellular senile plaque (SP) deposition and intraneuronal neurofibrillary tangles (NFTs) [3]. While the exact pathogenic contribution of these two AD hallmarks and their constituents, such as aggregation-prone amyloid β (Aβ) peptide species and hyperphosphorylated tau protein, remain unclear, a growing body of evidence suggests that their development may be paralleled or even preceded by alterations or dysfunctions in the endolysosomal and the autophagic systems [4].

Autophagy, a lysosome-mediated cell self-processing system, is closely associated with the elimination of abnormally accumulated AD-related proteins [5–7]. Macroautophagy is the most important form of autophagy [8, 9]. When cells receive autophagy-inducing signals such as hunger and growth factor deficiency, autophagy starts with the formation of autophagy-related protein complexes, and autophagosome (AP) with the bilayer membrane structure gradually formed, mainly around axons [10–12]. Mature APs are transported back to the cell body through the axonal microtubule system, then fuse with lysosomes which mainly located around the nucleus to form autolysosomes (ALs) with monolayer membranes, and then autophagy-related encapsulated proteins are released as a result of cathepsin (CTS) B and CTSD degradation. In their degradation, lysosomal acidification plays an important role [13]. Normally, APs can be quickly transported to the cell body and degraded, so APs are rarely seen in normal nervous tissue. However, as AD develops, the maturation and degradation of APs become blocked. The APs that aggregate in AD patients and model mice are rich in Amyloid precursor protein (APP), Aβ, β-secretase and γ-secretase complexes [13, 14]. Therefore, an abnormal increase in autophagy may result in the accumulation of APs and a decrease in the clearance of Aβ, resulting in a further increase in Aβ burden [15, 16]. In addition, the excessive phosphorylation of tau protein causes axonal transport defects, diminishing the effectiveness of the transport function and causing autophagic vesicle accumulation and inflammatory changes in nerve processes. The maturation of APs and their fusion with lysosomes are blocked, which will prevent the completion of autophagy, phosphorylated tau that cannot be degraded in time, and ALs easily aggregate to form NFTs [17–26]. Consequently, it has been reported that autophagy acts as a "double-edged sword" in the development of AD [27–35].

Autophagy flux, the rate at which long-lived protein aggregates are degraded by autophagy, involves the whole autophagy process, including the formation of APs, the transport of substrates to lysosomes, the degradation of substrates and the release of macromolecular substances back into the cytoplasm [36]. The study of the dynamic changes in autophagic flux throughout the course of AD is an important approach to reveal the pathogenesis of AD and find new targets for the prevention and treatment of AD. However, it is not clear which dysregulated step in the autophagic flux process plays a key role in the pathogenesis of AD. In this study, we studied changes in autophagic flux in AD by using brain tissue of postmortem AD patients, APP/PS1/LC3 triple-transgenic mice (3×TgAD mice) and SH-SY5Y cells stably expressing APPswe. These results suggest that AP-lysosome fusion is hindered, and dysregulated AL degradation increases Aβ levels. The aggregation of SPs may be the main outcome of dysregulated autophagy in AD. The increased Aβ, rather than overexpressed APPswe, activates autophagy. Both exogenous Aβ treatment and endogenous APPswe overexpression blocked AP-lysosome fusion and decreased lysosomal function in AD.

## RESULTS

### APs accumulation and the expression of lysosome-associated proteins and lysosomal enzymes is decreased in the brains of AD patients

**Figure 1:**
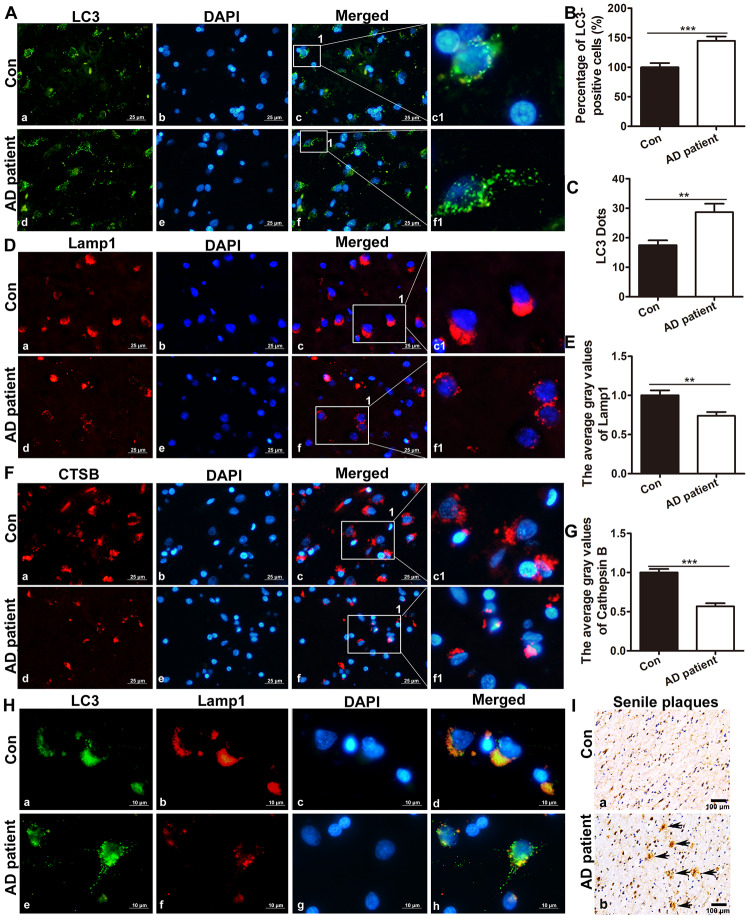
**The expression of LC3, Lamp1 and CTSB in the brain of postmortem patients.** (**A**) Immunofluorescence staining showed the expression of LC3 in the brain tissue of controls and AD patients (a, d: the expression of LC3; b, e: cell nuclei counterstained with DAPI; c, f: the merged images of Lamp1 and DAPI; c1 and f1 are partial magnifications of c and f. a-f: scale bar = 25 μm). (**B**) Quantitative analysis showing that the ratio of LC3-positive cells in the brain tissue. (**C**) Quantitative analysis showing the average LC3 puncta in brain tissue cells of AD patients and controls. (**D**) Immunofluorescence staining showing that the expression of Lamp1 in the brain tissue of controls (a-c1) and AD patients (d-f1). Scale bar = 25 μm. (**E**) Statistical analysis showing the average gray level of Lamp1. (**F**) Immunofluorescence staining showing that the expression of CTSB in the brain tissue of controls (a-c1) and AD patients (d-f1). Scale bar = 25 μm. (**G**) Statistical analysis showing the average gray level of CTSB. (**H**) Double immunofluorescence staining showing co-expression of autophagy- and lysosome-associated markers in the brain tissue of controls (a-d) and AD patients (c-f1). Scale bar = 10 μm. (**I**) Immunohistochemistry staining showing SPs in the postmortem cortexes of control patients (a) and AD patients (b), scale bar: 100 μm, the arrow indicates SP. The data are plotted as the mean ± SEM of three independent experiments and were analyzed by *t* test (**P < 0.01, ***P < 0.001 vs. control, n = 8).

Immunofluorescence staining of LC3 in the brain of postmortem patients showed that compared with that in the controls, the number of cells with active autophagy (LC3-positive cells among the total cells) in AD patients increased significantly by t – test (t = 4.383, P < 0.001), and the average gray level of LC3 expression in AD patients increased significantly (t = 3.397, P < 0.01). See Figure 1A–1C.

Immunofluorescence staining showed that the average gray level of Lamp1 expression in the brain tissue of AD patients was significantly lower than that in the controls (t = 3.238, P = 0.0016). The average gray level of CTSB in the brain tissue of AD patients also reduced significantly (t = 7.149, P < 0.001). See Figure 1D–1G.

Double immunofluorescence staining showed that the co-expression of the autophagy marker LC3 and the lysosomal marker Lamp1 was lower in AD patients than the controls, indicating the block in the fusion of APs and lysosomes, or the reduction in the number of lysosomes. (Figure 1H). Immunohistochemistry staining confirmed that there were many SPs in the postmortem cortex of AD patients, whereas no plaques were present in the postmortem cortex of the control patients (Figure 1I).

### Autophagic flux in the brains of APP/PS1 double-transgenic AD model mice

**Figure 2:**
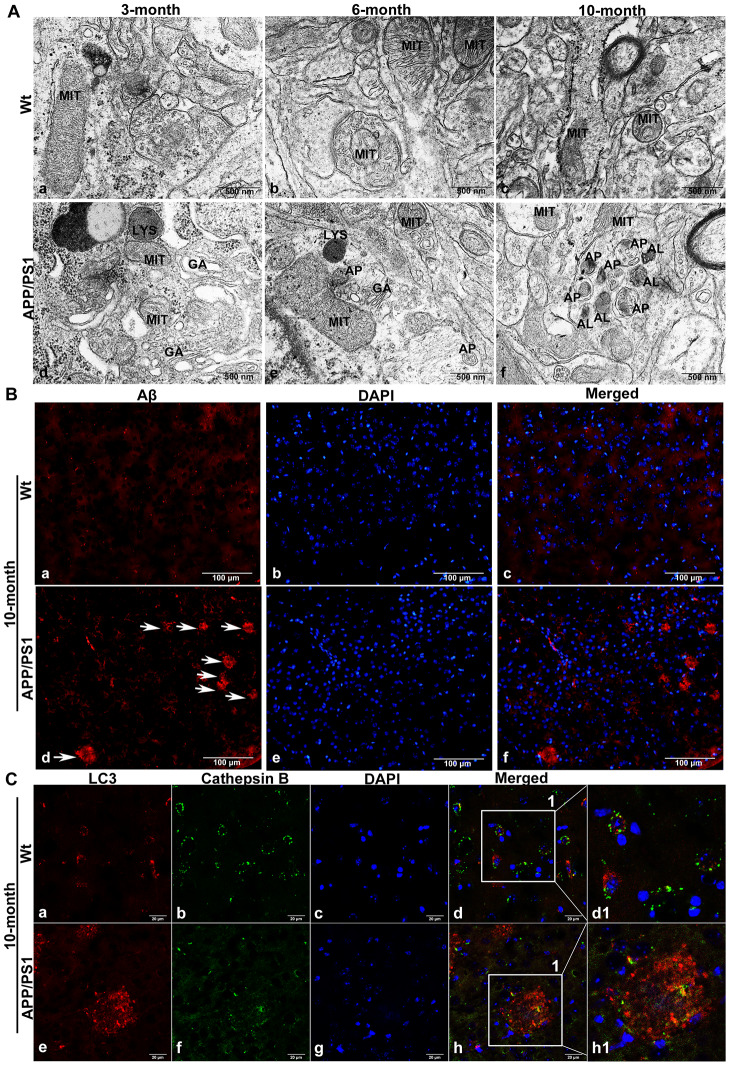
**The accumulation of APs in the brain tissue of APP/PS1 DTg AD mice.** (**A**) TEM showing little autophagy in wild-type (Wt) mice in the same litter (a-c); APs were also not easily observed in the brains of 3-month-old DTg mice (d); APs could be observed in the brains of 6-month-old DTg mice (e); a large number of APs and ALs had accumulated in the damaged axonal of brain in 10-month-old DTg mice (f). AL: autolysosome, AP: autophagosome, GA: Golgi apparatus, LYS: lysosome, MIT: mitochondria, Scale bar = 500 nm. (**B**) anti-Aβ 4G8 immunofluorescence staining showing no SPs in the cortex of the wild-type mice (a-c), while many SPs formed by the excessive accumulation of Aβ outside the cells in the cortex of DTg mice, (d-f, The arrow represents SP). Scale bar = 100 μm. (**C**) Double immunofluorescence staining showing that compared with that in Wt mice (a-d1), the expression of LC3 in 10-month-old APP/PS1 DTg mice increased significantly (e), the expression of CTSB decreased significantly (f), cell nuclei were counterstained with DAPI (g), and the co-expression of autophagosomal and lysosomal markers reduced (h). Scale bar = 20 μm, d1, h1 is a partial magnification of d and h.

Transmission electron microscope (TEM) results showed that it was difficult to observe APs in wild-type mice; APs were also not easily observed in the brains of 3-month-old APP/PS1 double-transgenic (DTg) AD model mice, while APs could be observed in the brains of 6-month-old DTg mice. In 10-month-old DTg mice, a large number of APs and ALs had accumulated in the damaged axonal of brain (Figure 2A).

Brain tissue sections of 10-month-old DTg mice were subjected to anti-Aβ 4G8 immunofluorescence staining and double immunofluorescence staining. The results showed that in the cortex of DTg mice, there were many SPs formed by the excessive accumulation of Aβ outside the cells, whose deposition lacked nuclei, while no SPs were present in the cortex of the wild-type mice (Figure 2B). Compared with the wild-type mice in the same litter, LC3 accumulated in the cortex where also lacked nuclei, lysosomal enzyme CTSB expression decreased significantly, and LC3 and CTSB co-expression decreased in the brain tissue of 10-month-old DTg mice (Figure 2C).

### Expression of autophagic flux related proteins in the brains of DTg AD model mice

**Figure 3:**
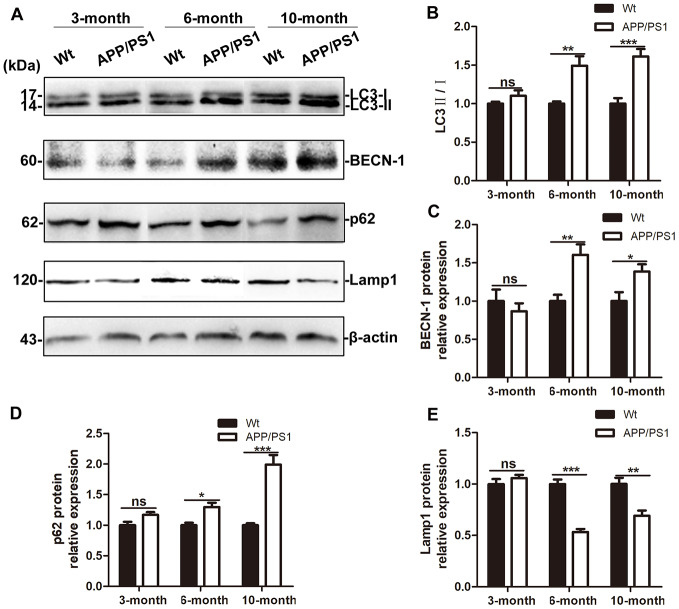
**Autophagic flux in the brains of APP/PS1 DTg AD mice of ages.** (**A**) Western blot showing LC3, BECN-1, p62 and Lamp1 expression in the brains of wild-type (Wt) and APP/PS1 DTg mice. (**B**–**E**) Relative gray density analysis of LC3-II/LC3-I, BECN-1, p62, and Lamp1 expression levels. The data are presented as the mean ± SEM and were analyzed by *t* test. (Compared with Wt mice, * P < 0.05, ** P < 0.01, *** < 0.001; “ns” denotes that there was no significant difference, n=3).

Western blot were used to assess the expression of autophagic flux - related protein. In 3-month-old DTg mice, the expression of LC3 was slightly increased but the difference is not statistically significant compared with that in the wild-type mice in the same litter (t = 1.358, P > 0.05); there were also no significant changes in BECN-1 protein, SQSTM1/p62 protein, or Lamp1 protein in the brain of DTg mice (t = 0.7326, 2.406, and 1.000; P > 0.05). In 6- and 10-month-old mice, compared with that in wild-type mice in the same litter, the expression of LC3 protein, BECN-1 protein and p62 protein in the brains of DTg mice all increased (LC3 in 6-month-old mice: t = 5.436, P < 0.01; LC3 in 10-month-old mice: t = 7.181, P < 0.001; BECN-1 in 6-month-old mice: t = 3.796, P < 0.01; BECN-1 in 10-month-old mice: t = 0.587, P < 0.05; p62 in 6-month-old mice: t = 3.670, P < 0.05, and p62 in 10-month-old mice: t = 6.142, P < 0.001), while the expression of Lamp1 protein in the brains of 6- and 10-month-old DTg mice decreased (t = 8.705, P < 0.001; t = 3.912, P < 0.01). See Figure 3A–3E.

### Expression of SP, AP and lysosomal markers in the brains of APP/PS1/LC3 autophagic flux AD model mice

**Figure 4:**
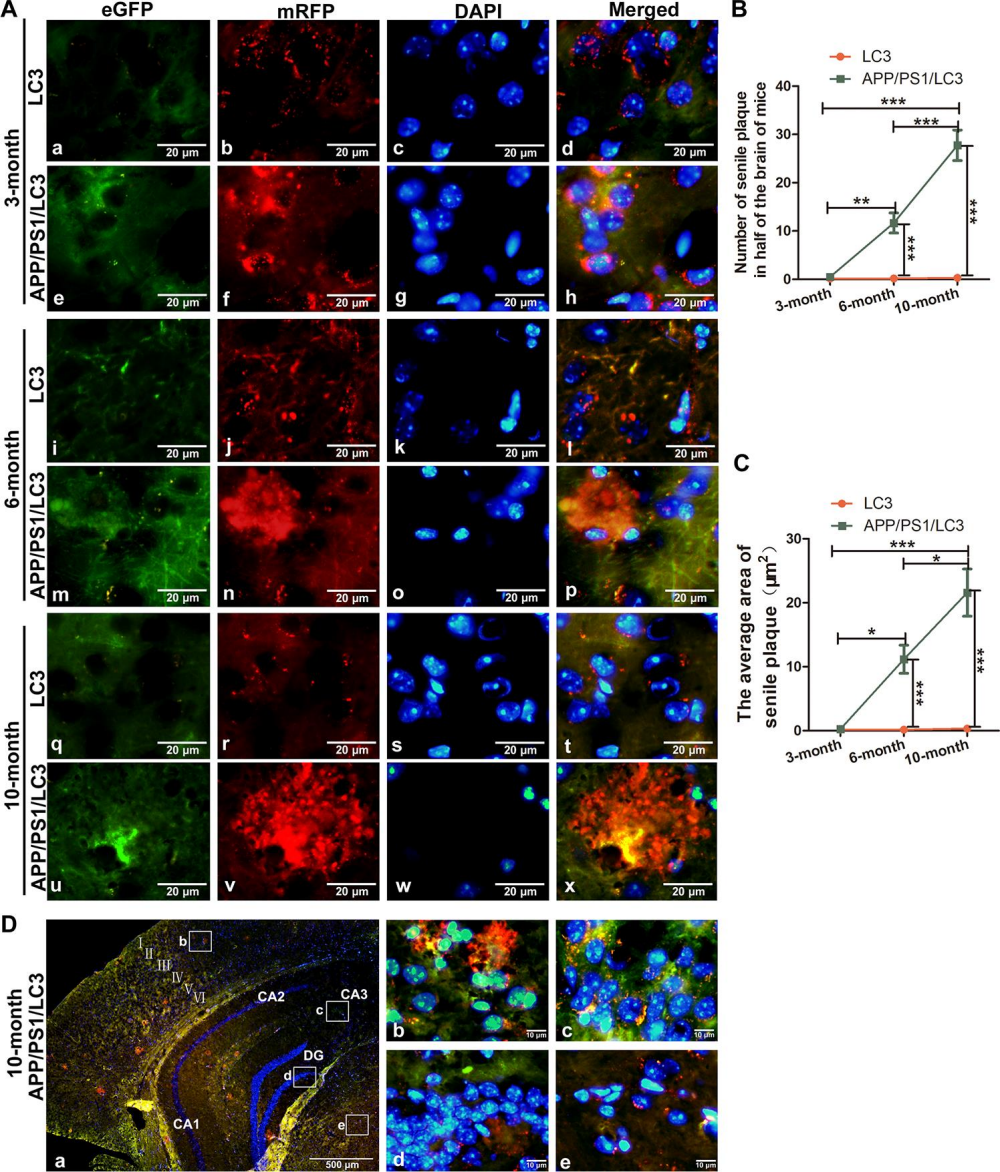
**The co-existence of APs, ALs and SPs in the brains of APP/PS1/LC3 autophagic flux AD model mice.** (**A**) In the brains of 3-month-old 3×Tg mice, the AP and AL proportion increased, but no SPs were found. In the brains of 6- and 10-month-old 3×Tg mice, SPs coexisted with APs and ALs. Scale bar = 20 μm. (**B** and **C**) The quantification of SPs in 3×Tg mice and littoral LC3 mice. (*P < 0.05, **P < 0.01, ***P < 0.001, n=3). (**D**) Autophagic flux in the different brain regions of 10-month-old 3×Tg mice. There were more APs but less ALs in the cortex and hippocampus, while there were less APs but more ALs in the white matter. Scale bar: a, 500 μm, b-e, 10 μm.

To evaluate the relationship between autophagic flux and the pathological products of AD, DTg AD model mice were housed with CAG-mRFP-GFP-LC3 transgenic mice to breed APP/PS1/LC3 transgenic mice (3×Tg mice), which are a useful AD model for evaluating autophagic flux. The APs and lysosomes in the brains of 3-, 6- and 10-month-old 3×Tg mice were observed. There were neither areas with high accumulation of APs and ALs, nor SP deposition in the brains of 3-month-old mice, while a lot of APs and ALs accumulated outside the cells in the cortex and hippocampus of 6- and 10-month-old 3×Tg mice. (Figure 4A–4C). In 10-month-old 3×Tg mice, there were more APs but less ALs in the cortex and hippocampus where prone to SP formation, while there were less APs but more ALs in the white matter where SPs do not easily form (Figure 4D).

**Figure 5:**
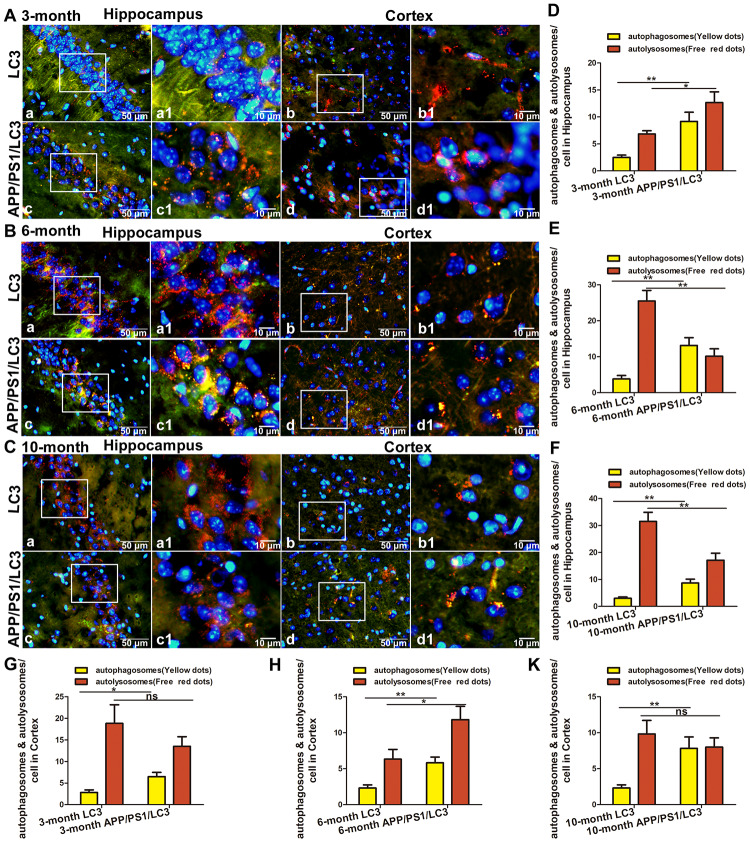
**The expression of AP and AL in the brains of APP/PS1/LC3 autophagic flux AD model mice.** (**A**–**C**) The APs and ALs in the hippocampus and cortex of 3, 6, 10 -month-old LC3 and 3×Tg mice. Scale bar: a-d, 50 μm, a1-d1, 10 μm, a and c: hippocampus, b and d: cortex, a1-d1 are partial magnifications of a-d. (**D**–**K**) The number of mRFP and GFP spots in 30 cells from 5 high-power fields was counted. The yellow dots of mRFP and GFP colocalization represent APs, and the free red dots represent ALs. (Compared with LC3 mice, *P<0.05, **P<0.01, ***P<0.001; “ns” denotes that there was no significant difference, n = 3).

In the hippocampus, the number of APs in the 3×Tg mice at 3-, 6- and 10-months old increased significantly, compared with that in the LC3 autophagic flux mice in the same litter (t = 3.800, 3.972, and 3.675, all P < 0.01). The proportion of ALs in the hippocampus of 3×Tg mice at 3 month-old increased significantly (t = 2.800, P < 0.05), whereas that decreased significantly in 3×Tg mice at 6 and 10 months of age (t = 3.994, 3.334, both P < 0.01). In the cortex, the number of APs in the 3×Tg mice increased significantly at 3-, 6- and 10-months old (t = 3.162, P < 0.05; t = 3.900, P < 0.01; and t = 3.365; P < 0.01), but there was no significant change in the AL number at 3 and 10 months of age (t = 1.095 and 0.8018; both P > 0.05), and a little increase in the AL number at 6 months of age (t = 2.773, P < 0.05). See Figure 5A–5K.

### Culture and treatment of SH-SY5Y cells and APP_swe_-overexpressing SH-SY5Y cells

**Figure 6:**
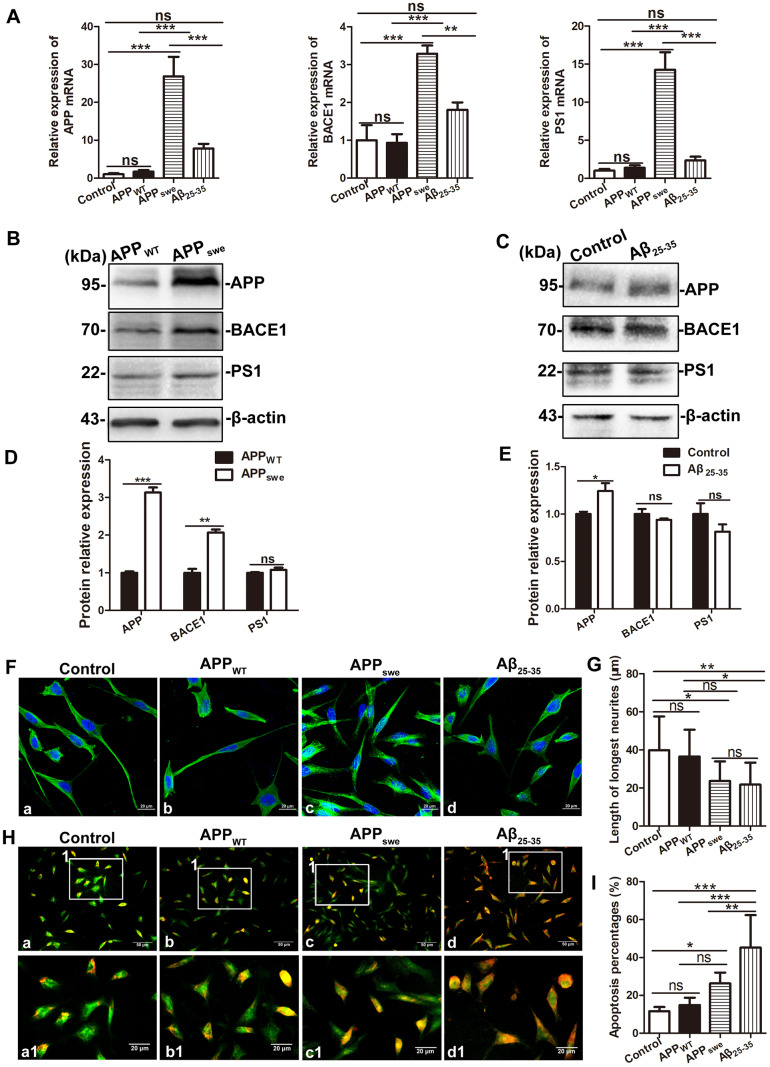
**Comparison of SH-SY5Y cells (Control), SH-SY5Y cells transfected with empty vector (APP_WT_), APPswe-overexpressing SH-SY5Y cells (APP_swe_) and SH-SY5Y cells treated with 30 μM Aβ_25-35_ (SH-SY5Y +Aβ _25-35_).** (**A**) Real-time PCR assay showing the expression of APP, BACE1 and PS1 mRNA in the four group cells. (**B** and **C**) Western blot showing the expression of APP, BACE1 and PS1 in APP_WT_ and APP_swe_ cells, SH-SY5Y cells and SH-SY5Y +Aβ _25-35_ cells. (**D** and **E**) Relative protein gray density analysis. (**F**) Tubulin staining showing the morphology of the four group cells. Scale bar = 20 μm. (**G**) Statistical analysis of the longest processes. (**H**) Acridine orange staining showing membrane stability of the four group cells. a –c: Scale bar = 50 μm, a1 – c1: Scale bar = 20 μm. (**I**) Statistical analysis of the apoptosis rate. (* P < 0.05, ** P < 0.01, *** P < 0.001; “ns” denotes that there was no significant difference, n=3).

We cultured SH-SY5Y cells and APP_swe_-overexpressing SH-SY5Y cells (APP_swe_). The real-time PCR results showed that compared with those in blank control SH-SY5Y cells (Control) and SH-SY5Y cells transfected with empty vector (APP_WT_), the levels of APP, BACE1, PS1 mRNA in APP_swe_ cells were significantly increased (APP_swe_ and Control: q = 9.659, 8.355, and 12.24, all P < 0.0001; APP_swe_ and APP_WT_: q = 9.394, 8.607, 11.91, all P < 0.001). Then SH-SY5Y cells were treated with 30 μM Aβ_25-35_ (Aβ_25-35_), the level of APP, BACE1 and PS1 mRNA in SH-SY5Y + Aβ_25-35_ cells did not change significantly (q = 2.550, 2.935 and 1.225, all P > 0.05). See Figure 6A.

Western blot analysis showed that the APP and BACE1 protein levels were increased in APP_swe_ cells compared with APP_WT_ cells (t = 15.62, P < 0.001; t = 8.275, P < 0.001), the level of PS1 protein level did not change significantly (t = 1.294, P > 0.05). The level of APP protein in SH-SY5Y + Aβ_25-35_ cells was slightly higher than that in SH-SY5Y cells (t = 2.823, P < 0.05). There were no significant changes in the levels of BACE1 and PS1 protein between SH-SY5Y and SH-SY5Y + Aβ_25-35_ cells (t = 1.579 and 1.355; both P > 0.05). See Figure 6B–6E.

Tubulin staining was used to assess the morphology of four group cells. After one-way ANOVA, Tukey's multiple comparison test showed that the longest neurites of APP_swe_ cells were shorter than those of control cells (q = 4.566, P < 0.05), while there was no significant difference between APP_WT_ cells and APP_swe_ cells (q = 3.627, P > 0.05). The longest neurites of SH-SY5Y + Aβ_25-35_ cells were shorter than those of SH-SY5Y control cells (q = 5.112, P < 0.01). Acridine orange staining was used to assess the cell membrane stability. Compared with that of control cells, the apoptosis rate of APP_swe_ cells was slightly increased (q = 4.468, P < 0.05), while there was no significant difference between APP_WT_ cells and APP_swe_ cells (q = 3.469, P > 0.05). And the apoptosis rate of SH-SY5Y + Aβ_25-35_ cells was significantly increased than that of control (q = 10.24, P < 0.001). See Figure 6F–6I.

### Effects of exogenous Aβ treatment and endogenous APP_swe_ overexpression on autophagic flux in AD model cells

**Figure 7:**
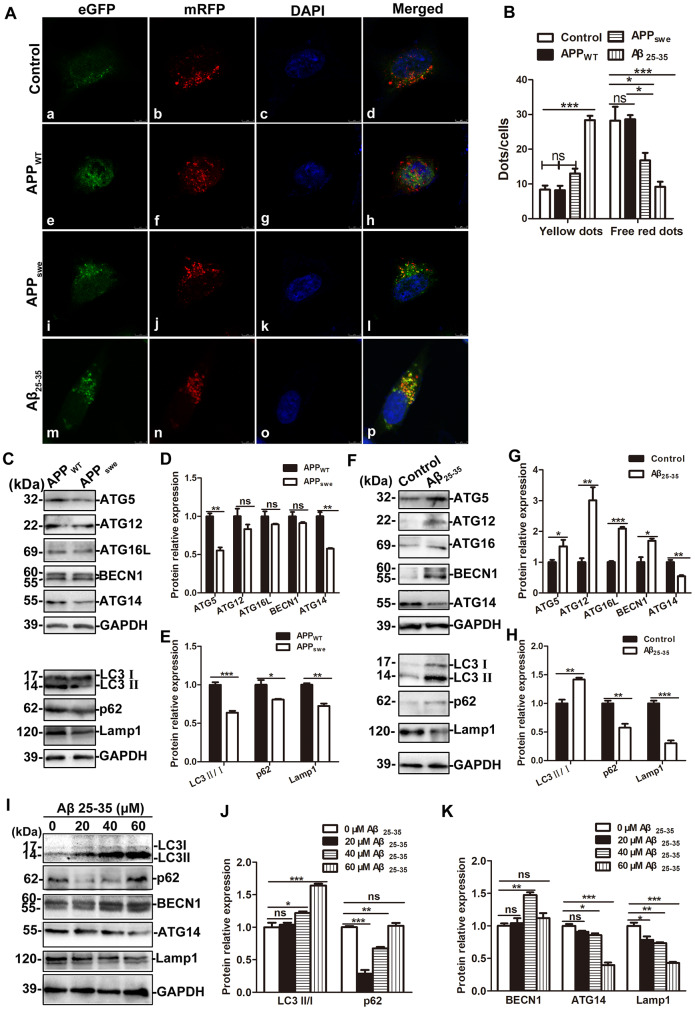
**The effects of exogenous Aβ _25-35_ treatment and endogenous overexpression of APP_swe_ on autophagic flux.** (**A**) Fluorescence microscopy images of control SH-SY5Y, APP_WT_, APP_swe_ and SH-SY5Y +Aβ _25-35_ (30 μM) cells infected with mRFP-GFP-LC3 adenovirus for 24 h and then treated with 100 nM rapamycin for 10 min (scale bar, 10 μm). (**B**) mRFP and GFP puncta were quantified to determine the number of APs and ALs per cell. For each group, 30 cells from 5 visual fields were randomly selected for counting (*P < 0.05, **P < 0.01, ***P < 0.001). (**C**) Western blot showing the expression of ATG5, ATG12, ATG16L, BECN1, ATG14, LC3, p62 and Lamp1 in APP_WT_ and APP_swe_ cells. (**D** and **E**) Relative protein gray density analysis. (**F**) Western blot analysis showing the expression of ATG5, ATG12, ATG16L, BECN1, ATG14, LC3, p62 and Lamp1 in SH-SY5Y and SH-SY5Y +Aβ _25-35_ (30 μM) cells. (**G** and **H**) Protein band relative gray density analysis. (**I**) Different concentrations of Aβ _25-35_ (0, 20, 40, and 60 μM) were used to treat SH-SY5Y cells for 24 h and the expression of LC3, p62, BECN1, ATG14 and Lamp1 were assessed by Western blot. (**J** and **K**) Relative protein gray density analysis. (* P < 0.05, ** P < 0.01, *** P < 0.001; “ns” denotes that there was no significant difference, n = 3).

To evaluate autophagic flux, cells were infected with adenovirus containing a dual red fluorescent protein–green fluorescent protein–microtubule-associated protein 1 light chain 3 (mRFP–GFP–LC3) for 24 h to detect autophagic flux. We then evaluated the formation of AP and AL by counting fluorescent puncta. When autophagy was activated by 100 nM rapamycin for 10 min, GFP and mRFP dots could be observed clearly. The co-localization GFP and mRFP were observed as yellow dots, which represent APs and partially acidified ALs. The free red staining represents fully acidified ALs [40]. Compared with the control cells and APP_WT_ cells, the yellow dots in APP_swe_ cells did not change significantly (q = 3.620, 3.777, both P > 0.05), but the free red dots decreased slightly (q = 4.618, 4.780, both P < 0.05), while compared with the control cells, the number of yellow dots in the SH-SY5Y + Aβ_25-35_ cells increased significantly (q = 15.74, P < 0.001), and the number of free red dots decreased markedly (q = 7.696, P < 0.001). See Figure 7A, 7B.

Western blot results showed that compared with those in APP_WT_ cells, the levels of ATG5 and ATG14 in APP_swe_ cells decreased significantly (t = 7.922, 7.800, P < 0.01), and the levels of ATG12, ATG16L and BECN-1 protein were not significantly changed (t = 1.442, 1.626, and 1.530, P > 0.05). The levels of LC3, p62 and Lamp1 protein decreased (t = 8.660, P < 0.001; t = 3.083, P < 0.05; t = 7.463, P < 0.01, Figure 7C–7E). Compared with control cells, the expression of proteins associated with the early stage of autophagic flux such as ATG5, ATG12, ATG16L and BECN-1 upregulated in SH-SY5Y + Aβ_25-35_ cells (t = 2.328, P < 0.05; t = 4.547, P < 0.01; t = 16.81, P < 0.001; t = 3.904, P < 0.05); Furthermore, the expression of LC3II/I upregulated (t = 5.801, P < 0.01), the expression of p62 protein downregulated (t=5.176, P < 0.01), and the expression of mid- and late-stage-associated autophagic flux proteins such as ATG14 and Lamp1 downregulated significantly (t = 5.757, P < 0.01; t =10.43; P < 0.001; Figure 7F–7H).

Then, SH-SY5Y cells were treated with different concentrations of Aβ _25-35_ (0, 20, 40, and 60 μM) for 24 h, and the cells were collected to extract proteins for Western blot test. The results showed that as the Aβ _25-35_ concentration increased, the expression of LC3II/I increased gradually (q = 0.8595, P > 0.05; q = 5.013, P < 0.05; q = 14.830, P < 0.001); the expression of p62 protein initially decreased and then increased (q =18.870, P < 0.001; q = 8.620, P < 0.01; q = 0.562, P > 0.05); BECN-1 expression initially increased and then decreased (q = 0.662, P > 0.05; q = 7.635, P < 0.001; q =1.920, P > 0.05), and ATG14 and Lamp1 expression decreased gradually (ATG14: q = 3.096, P > 0.05; q =4.899, P < 0.05; q = 15.870, P < 0.001; Lamp1: q = 5.706, P < 0.05; q = 7.018, P < 0.01; q = 15.150, P < 0.001). See Figure 7I–7K.

## DISCUSSION

Autophagy is closely associated with neurodegenerative diseases such as AD. Autophagic flux is a new concept proposed in recent years, which includes the entire autophagy process, including the formation of APs, the transport of substrates to lysosomes, the degradation of substrates and the release of macromolecular substances back into the cytoplasm [6, 7]. To study the role of autophagy in the pathogenesis of AD, the process of autophagic flux needs to be monitored dynamically, which is an important approach to reveal the pathogenesis of AD.

It was reported that there are a large number of lysosomes in the brain tissue of patients with AD, but there are also a large number of immature autophagic vesicles around the abundant lysosomes, as well as cathepsin-immunoreactive substances and many substrates in the APs [37]. In this study, it was found that the expression of LC3 in the brain tissue of postmortem AD patients increased significantly, indicating that there were a large number of APs or ALs in the brains of the AD patients. However, the increase in APs may be caused by the activation of autophagy on the one hand and the reduced degradation of ALs on the other hand. Further detection results showed that the expression of the lysosomal markers Lamp1 and CTSB decreased significantly, and the co-localization of LC3 and Lamp1 reduced in the AD group than the control group, suggesting the block in the fusion of APs and lysosomes, or the decrease of lysosome number and function. This result is not consistent with that of Nixon's report and may be related to the age of the AD patients and the course of AD. Studies have shown that autophagy in the brain of AD patients is closely associated with the development of the disease [38]. In the early stage of AD, autophagy can accelerate the clearance of denatured protein and promote the survival of neurons. As AD develops, an increasing number of pathological proteins accumulate, the number of lysosomes decreases, the rate of autophagy decreases, the clearance rate of Aβ and tau protein decreases, and finally, apoptosis or autophagic cell death can be induced. In this study, the AD patients, who were 70-101 years old, were mostly in the mid and late stages of AD. They had obvious SPs deposition in the brain. Therefore, autophagy in the brains of AD patients increased obviously, but lysosome generally decreased.

In order to further explore the exact relationship between autophagic flux and the development of AD, different age of APP / PS1 DTg mice were studied. Two-to-three-month old DTg mice began to show increased soluble Aβ levels, but no SPs formation, which can indicate the pre-onset stage of AD. Five-to-six-month old DTg mice had SPs in the brain and behavioral abnormalities, which can represent the early stage of AD. Nine-to-ten-month old DTg mice had many SPs in the brain and an increased number of behavioral abnormalities, which usually, represent the mid and late stages of AD [39]. In this study, the result indicated that APs did not accumulate before the formation of SPs but accumulated along with the deposition of SPs in the brain of DTg mice. The SPs are formed by the excessive accumulation of Aβ outside the cells, and the area of the SPs deposition lacks nuclei. In the brains of 10-month old DTg mice, LC3 expression accumulated markedly and there were nearly no nuclei around the same area, suggesting LC3 may be highly expressed in the SP area.

Then, the expression of autophagic flux-related proteins were detected in the brains of DTg mice. When autophagy occurs, LC3 changes from type I to type II, but the ratio of LC3 II / I increases; this increase can be caused by autophagy activation or reduced AL degradation [40]. BECN-1 is a key factor in the early regulation of autophagy and is indispensable for AP formation [41]. As a lysosomal degradation substrate, SQSTM1/p62 protein levels decrease when autophagy occurs and progresses normally. When autophagy activity is blocked, p62 accumulates continuously [42, 43]. In this study, the expression of LC3 protein, BECN-1 protein and p62 protein in the brains of 6- and 10-month-old DTg mice increased, indicating that as the age of DTg mice increases, the autophagic flux degrades in response to the formation of SPs. Autophagy is a process in which the components of cells are degraded in lysosomes. The degradation of AD pathological proteins by lysosomes is influenced by the number of lysosomes, the lysosomal pH, the content and activity of proteases in lysosomes and other factors [44, 45]. It has been suggested that the main autophagy pathway defect in the brains of AD patients is the dysregulation of autophagolysosomal proteolysis [46]. In AD model mice, the expression of CSTB decreased significantly, and the co-expression of LC3 and CSTB reduced, which is consistent with the human brain tissue detection result, indicating that the lysosomal degradation function decreased in AD and may be the main reason why autophagy aggravates the progression of AD.

In order to further explore the relationship between SPs and autophagic flux, 3×Tg mice which contain the CAG-mRFP-eGFP-LC3 gene and APP / PS1 gene were studied. This kind of mice can simulate the main pathological characteristics and behavioral disorders of AD, and also can be used to measure autophagosomal-lysosomal transport. When APs are formed, the mRFP-eGFP-LC3 fusion protein localizes to the AP membrane forming yellow fluorescent spots under a fluorescence microscope, representing APs and ALs with minimal acidification. The autophagy process can be evaluated by the ratio of GFP to mRFP [47]. The results showed that there were neither areas with high accumulation of APs and ALs, nor SP deposition in the brains of 3-month-old mice, while a lot of APs and ALs accumulated outside the cells in the cortex and hippocampus of 6- and 10-month-old mice. We previously have found that this 3×Tg mice began to show SP formation at the age of 6 months, and SPs are very obvious at the age of 10 months. Take 10-month old 3×Tg mice for instance, there were more APs but less ALs in the cortex and hippocampus where prone to SP formation, suggesting the block in the fusion of APs and lysosomes, while there were less APs but more ALs in the white matter where SPs do not easily form, suggesting autophagic flux works well around the same area. The number of APs in the brains of 3×Tg mice increased with age, but the number of ALs did not increase accordingly. The increase in APs could be caused by an increase in autophagy activation or APs degradation reduction, and the decrease in ALs could be caused by the inhibition of fusion between APs and lysosomes. In the cortex of 6-month old 3×Tg mice, with the appearance of SPs and the activation of autophagy, APs increased and ALs also increased correspondingly, which may enhance the degradation of abnormal proteins; while in the cortex of 10-month old 3×Tg mice, with more significant accumulation of SPs, APs increased and ALs began to decrease, indicating the block in the fusion of autophagy and lysosome, or the decrease of lysosome number. However, in the hippocampus, APs increase and ALs decreased both in 6-month old and 10-month old 3×Tg mice, suggesting that the fusion and degradation of APs and lysosome in the hippocampus may occur earlier than in the cortex of AD model mice.

The SPs are formed by a large amount of extracellular Aβ, which is mainly produced by the abnormal shearing of APP by β - secretase and γ - secretase, respectively. In order to further explore the effects of APP mutations and Aβ on autophagic flux, SH-SY5Y cells stably expressing the mutant gene APP_swe_ were constructed. The real-time PCR and Western blot results showed that the levels of APP, BACE1 (the core component of β - secretase) and PS1 (the core component of γ - secretase) mRNA and protein increased in APP_swe_ cells, while did not change significantly in SH-SY5Y cells treated with 30 μM Aβ_25-35_. Then, Acridine orange staining was used to detect apoptosis [48]. The finding suggests that a certain concentration of Aβ is more likely to cause SH-SY5Y apoptosis than the mutant APP gene.

In order to further evaluate the autophagic flux, the formation of APs and ALs were evaluated by fluorescent puncta counting 24 h after infection with the mRFP-eGFP-LC3 adenovirus. The co-localization of mRFP and GFP dots produces yellow dots, representing APs and ALs with minimal acidification. Free red mRFP dots represent fully acidified ALs [47]. When autophagy was activated by rapamycin, the number of APs and minimally acidified ALs in the stable APPswe-expressing cells did not change significantly, whereas that in SH-SY5Y cells treated with Aβ_25-35_ increased significantly. The number of fully acidified ALs in both the APP_swe_ cells and cells treated with Aβ_25-35_ decreased markedly, which proved that the increase in Aβ_25-35_ rather than the overexpression of APP_swe_ activated autophagy. The effects of exogenous Aβ_25-35_ treatment and endogenous APP_swe_ overexpression on autophagic flux in AD model cells were further detect. The markers such as ATG5, ATG12, ATG16L and BECN-1 are involved in the early stage of autophagy, ATG14 has been shown to form a complex with BECN-1 in the early stage of autophagy and promote the fusion of APs and lysosomes in the middle stage of autophagy [49], Lamp1 and CTSB are markers of the late stage of autophagy. The results showed that the levels of ATG5, ATG14, LC3, p62, and Lamp1 protein in APP_swe_ cells reduced notably, indicating that the overexpression of APP_swe_ did not activate autophagy, but blocked the autophagic flux. Nevertheless, exogenous Aβ_25-35_ treatment upregulated the expression of proteins involved in the early stage of autophagy, reduced the level of the markers in the middle and late stage of autophagy, indicating that a certain concentration of Aβ can activate autophagy, blocked the fusion of APs and lysosomes, and inhibit lysosomal function.

In conclusion, in postmortem AD patients, LC3 increased, whereas the expression of the lysosomal marker Lamp1 and CSTB decreased significantly, and the co-localization of APs and lysosomes decreased. In the brains of AD transgenic mice without SPs, the APs and ALs increased, and the fusion of APs with lysosomes is unaltered. However, in the brains of AD transgenic mice with SPs, APs increased and the expression of CSTB decreased significantly. The activation of autophagy is mainly due to the increase in Aβ rather than the mutation of the APP gene. However, both the treatment with exogenous Aβ_25-35_ and the mutation of the endogenous APP gene block the fusion of APs with lysosomes in AD model cells, which is the main defect in the autophagy pathway. The results of this study further clarify the relationship between autophagic flux and AD, which could provide a new method to improve autophagic flux for the treatment of AD.

## MATERIALS AND METHODS

### Postmortem human brain and tissue preparation

Postmortem human brains were collected through the willed body donation program, which exists with government (municipal police department and office of the Red Cross Society of China) and university approval to provide cadavers for teaching anatomy to medical students. Efforts are being taken to develop this platform for the human brain bank to fuel the initiatives supporting the China Brain Project. Brain tissue was obtained from the human brain bank at Xiangya University. Samples from clinically diagnosed AD patients and age-matched non-AD subjects were selected for use in the present study (Table 1). Brains were evaluated for AD neuropathological changes, which were scored using Braak’s staging system; cases were selected and grouped into the AD group that had a clinical history of dementia, with Braak’s scores of postmortem neuropathology at or greater than IV. Thus, all cases in the control group were selected based on the fact that they showed no or only mild amyloid and/or tau pathology in the cortex, with neuropathology limited to Braak’s stages I–II. The neuropathological characterization of some of the postmortem human brains has been described in previously published studies [50–52]. All human brains were banked according to a standardized procedure with one side of each brain rapidly frozen and stored at -70°C (for future biochemical studies), and the other side immersion fixed in 10% formalin for anatomical/pathological studies. In the present study, sections from a slice of the mid-hippocampal temporal lobe (~2 cm in thickness) were used. This temporal lobe block was removed from the formalin-fixed hemisphere, cryoprotected in 30% sucrose, then, 20 μ m thick coronal sections were cut by frozen microtome (Leica Instruments, Germany). The sections were then stored in a cryoprotectant at -20°C until being processed histologically.

**Table 1:** Patient information, grouping and Braak scoring.

**Group case**	**Code**	**Postmortem delay (h)**	**Amyloid plaques**	**Neurofibrillary tangles**
Group with brain amyloid pathology (male: 9, female: 1, age range: 70-101 years)	Case1	4.5	C	V
	Case2	6.3	C	IV
	Case3	36	C	I
	Case4	9.5	C	III-VI
	Case5	8	C	II
	Case6	18	C	III
	Case7	25	C	II
	Case8	54	C	III-IV
Control group (male: 6, female: 1, age range: 64-88 years)	Case1	5.3	N/A	N/A
	Case2	6	N/A	I
	Case3	26.5	N/A	N/A
	Case4	16.5	N/A	N/A
	Case5	18	N/A	N/A
	Case6	18	N/A	N/A
	Case7	6	N/A	N/A
	Case8	13.5	N/A	N/A
		P=0.285	N/A: not applicable	

### Immunohistochemistry and Immunofluorescence

Four sets of consecutive mid-hippocampal temporal lobe sections from each postmortem human brain were stained immunohistochemically with antibodies: monoclonal mouse anti-Aβ 6E10 (1:5000, #39320, Signet Laboratories Inc., Dedham, MA, USA). Several section pretreatments were used, including sortilin immunogenic peptide blocking (at 5- and 10-times the primary antibody concentration), heating (65°C), and formic acid (100%) and guanidine hydrochloride (HCl; 5 M) treatment. Next, all sections were treated free-floating with 5% H_2_O_2_ in PBS for 30 min and 5% normal horse serum in PBS with 0.3% Triton X-100 for 1 h, followed by incubation with primary antibodie 6E10 at 4°C overnight. The sections were then incubated with biotinylated horse anti-mouse IgGs at 1:400 for 1 h and ABC reagents (1:400) for 1 h. Plaques were visualized by the DAB method and counted by microscopy.

anti-Aβ immunofluorescence staining was initiated with sections pretreated with formic acid for 1 h at room temperature, then washed with 0.01 M PBS 3 times and blocked with 5% fetal calf serum in 0.3% Triton X-100 for 30 min at 37°C. Next, the sections were soaked in 0.1% Triton and 3% H_2_O_2_ in 0.01 M PBS for 30 minutes at 95 °C by using water bath. Then, the sections were incubated with the primary antibody 4G8 (1:200 dilution; Biolegend, USA) overnight at 4°C. The following day, bound primary antibodies were tagged with the Cy3-conjugatedsecondary antibody (Beyotime, Shanghai, China) for 30 min at 37°C. After immunofluorescence labeling, nuclei were visualized by DAPI (Beyotime, Shanghai, China) staining. Finally, the sections were covered with coverslips for observation with a fluorescence microscope (Leica, Germany).

Double immunofluorescence staining was initiated with section pretreatment in PBS containing 5% donkey serum for 30 min. The sections were then incubated overnight at 4°C with mouse anti-LC3 (1:500 dilution, Millipore, USA), together with one of the following: (1) rabbit anti-Lamp1 (ab24170, 1:200); (2) rabbit anti-CTSB (1:500 dilution, Cell Signaling Technology, USA). The antigens were detected with specific fluorescein-conjugated secondary antibodies (TRITC- or FITC-conjugated goat anti-mouse or goat anti-rabbit) for 1 h at room temperature. Then, the nuclei were counterstained with DAPI at room temperature. Images were collected and analyzed with a TCS-TIV confocal laser scanning microscope (Leica).

### Transgenic mice

Male APPswe/PS1ΔE9 DTg mice were purchased from the Institute of Laboratory Animal Sciences, Chinese Academy of Medical Sciences (Beijing, China). Female CAG-mRFP-GFP-LC3 transgenic mice were developed by Beijing CasGene Biotech Co., Ltd. One male DTg AD model mouse was caged with one-to-three female autophagic flux model mice for breeding. Animal care and experimental procedures were conducted according to the National Institutes of Health Guidelines for the Care and Use of Laboratory Animals and the Chongqing Medical University Policies for Animal Use. When the mouse offspring reached 3-weeks old, they were genotyped PCR using DNA extracted from tail tissues. A DNA Extraction kit (Invitrogen, USA) was used to extract genomic DNA according to the manufacturer’s instructions. The newly extracted DNA templates were further amplified by Platinum Tag DNA polymerase (Invitrogen, USA) in a 25 μl reaction volume. The following primers were used to specifically amplify the APP, PS1, and CAG genes: APP, forward 5'-CGG AAT TCC CTT GGT GTT CTT TGC AGA AG-3' and reverse 5'-CGG AAT TCC GTT CTG CAT CTC TCA AAG-3'; PS1, forward 5'-GGA TCC GCC ACC ATG GTG TGG TTG GTG AAT ATG GC-3' and reverse 5'-CGG GAT CCC TAG ATA TAA AAT TGA TGG-3', and CAG, forward 5’-GGCTC GTTTC TTTTC TGTGG CTG-3’ and reverse 5’- CCGAG GCTGG AGATG GAGAA GG-3’. Actin was used as an internal control. The samples were analyzed on a 1.2% agarose gel. The samples that presented APP, PS1, and CAG bands were from the 3×Tg mice.

### Transmission electron microscopy

For TEM analysis, 4 mice from each group were deeply anesthetized with an intraperitoneal injection of sodium pentobarbital (80 mg/kg nembutal) and perfused transcardially with 10 ml of phosphate-buffered saline (PBS) followed by 2.5% glutaraldehyde-4% paraformaldehyde in 0.01 M phosphate buffer (PB). The brain was quickly stripped in an ice bath, and tissues of 1 mm^3^ were cut from the hippocampal CA1 area. Then, the tissues were fixed in 2.5% electronic microscopy-grade glutaraldehyde for 2 h, washed several times with 0.01 M PBS, postfixed in 1% osmium tetroxide in 0.01 M PB for 2 h and dehydrated with a gradient alcohol series. Samples were embedded in Epon812 epoxy resin. Tissue blocks were then cut into 1 μm semithin sections, placed on slides, stained with azure-methylene blue and assessed by a light microscope. Then, areas were selected from the semithin sections and cut into thin sections. After uranyl acetate/lead citrate double staining, neurons and ultrastructures were observed on a Philips EM208S transmission electron microscope (Philips EM208S).

### Cell culture

SH-SY5Y cells and SH-SY5Y cells stably expressing APP_swe_ were donated by Professor Weihong Song at the University of British Columbia. The Swedish mutant APP (APPswe) was transfected into SH-SY5Y cells which were selected with antibiotics G418 (Sigma Co., St. Louis, MO, USA) and maintained in lab. These cell lines were cultured at 37°C with 5% CO_2_ in DMEM (HyClone Co., South Logan, VT, USA) containing 10% fetal calf serum (Gibco Co., Grand Island, NY, USA). The cells were divided into SH-SY5Y control group, APP_WT_ group (SH-SY5Y cells transfected with empty vector), APPswe group, and SH-SY5Y + A β_25-35_ group (SH-SY5Y cells treated with 30 μM A β _25-35_ for 24h). Acridine orange staining or β-Tubulin staining (rabbit anti β – Tubulin, 1:200 dilution, Cell Signaling Technology, USA) were performed after the cultured cells were fixed. The apoptosis rate was calculated and the neurite length were measured by Image J software.

### Infection with the mRFP–GFP–LC3 adenoviral Expression vector and Evaluation of fluorescent LC3 puncta

The cells were cultured on coverslips and transduced with mRFP–GFP–LC3 Adenovirus vector at an MOI of 50. Twenty-four hours after adenovirus transduction, the cells were treated with 100 nM rapamycin for 10 minutes, then washed with PBS, fixed with 4% paraformaldehyde, mounted with a reagent containing 40,6-diamidino-2-phenylindole (DAPI; Vectashield; Vector Laboratories, Inc.), and viewed by fluorescence microscopy (Nikon Eclipse E800). Images were captured on a Zeiss Laser Scanning Confocal Microscope (Carl Zeiss Inc., Jena, Germany). The number of GFP and mRFP puncta was determined by manual counting of fluorescent puncta in five fields. The number of nuclei was evaluated by counting the number of DAPI-stained nuclei in the same field. The number of puncta per cell was obtained by dividing the total number of dots by the number of nuclei in each microscopic field.

For the in vivo determination of the number of fluorescent LC3 puncta, fresh brain slices were embedded in Tissue-Tek OCT compound (Sakura FineTechnical Co., Ltd.) and frozen at -20°C. Sections 10 mm thick were obtained from the frozen tissue samples by using a cryostat (CM3050 S; Leica), air-dried for 30 min, fixed by washing in 95% ethanol for 10 min, mounted using a reagent containing DAPI, and viewed by fluorescence microscopy.

### Quantitative PCR

Total RNA was extracted from four group cells with TRI Reagent (T9424) following the manufacturer’s instructions. Reverse transcription was then performed using random hexamer primer and MMLV Reverse Transcriptase (M5301, Promega). Quantitative PCR (qPCR) for the quantitation of gene transcripts was performed with 2×HotStart SYBR Green qPCR Master Mix (TransGen Biotech, Beijing, China) using a Stratagene Mx3000P (Agilent Technologies). The primer sequences are as below. APP, forward: 5'- TGG CCC TGG AGA ACT ACA TC - 3’, and reverse: 5′- AAT CAC ACG GAG GTG TGT CA - 3′; BACE1, sense: 5 ′ - TCT GTC GGA GGG AGC ATG AT - 3′, and reverse:5 ′ -GCA AAC GAA GGT TGG TGG T - 3′; PS1, sense: 5’- CAT CAT GAT CAG TGT CAT TGT TGT - 3’; PS1, and reverse: 5’- TGC ATT ATA CTT GGA ATT TTT GGA - 3’ [53].

### Western blot analysis

Half of the brain tissues or cells were lysed in RIPA lysis buffer (Pierce, USA). The supernatants were separated by centrifugation at 14,000 x g for 15 min at 4°C. The protein concentration of the supernatant was determined by a Bio-Rad protein assay, and an equal amount of protein (50 μg) per lane was loaded on a 10% Tris-glycine SDS-PAGE gel. The electrophoresed proteins were transferred to PVDF (Millipore) membranes. The membranes were blocked in 5% BSA (dissolved in double-deionized water) and then sequentially incubated with mouse anti-LC3 (1:1000 dilution, Millipore, USA), rabbit anti-p62 (D5E2, 8025) and anti-CTSB (D1C7Y, 31718; 1:1000 dilution, Cell Signaling Technology, USA), rabbit anti-APP (ab32136), rabbit anti-BECN-1 (ab62557), rabbit anti-BACE1 (ab2077), rabbit anti-PS1 (ab71181), rabbit anti-Lamp1 (ab24170), mouse anti-amyloid precursor protein (ab32137), all 1:1000 dilution, Abcam, USA, rabbit anti-ATG5(DF6010), rabbit anti-ATG12(DF7937), and rabbit anti-ATG16L(DF3825), all 1:1000 dilution, Affinity, USA, rabbit anti-ATG14 (NBP2-36445, 1:1000 dilution, Novus), rabbit anti-GAPDH(AF7021, Affinity) and mouse anti-β-actin (A5441, Sigma Aldeich, 1:10000) antibodies overnight. The membranes were washed and then incubated with a horseradish peroxidase-conjugated secondary antibody. Immunoreactive bands were visualized by enhanced chemiluminescent detection (ECL, Amersham Pharmacia Biotech) using a Gel Imaging System (Bio-Rad, USA) and quantified with Quantity One image analysis software (Bio-Rad, USA).

### Statistical analysis

Data were analyzed using Graph Pad Prism 5 software. All values were expressed as the mean ± standard deviation (SD). The measurement data between the two groups were analyzed by t-test, and that among multiple groups were tested by analysis of variance (ANOVA) followed by Tukey's multiple comparison test. P values < 0.05 were considered significant.

### Ethics approval

All procedures involving animals were performed under institutional guidelines. This study was approved by The Ethics Committee of Chongqing Medical University (SCXK 2014-0004).
